# Development and characteristics of the Provincial Overdose Cohort in British Columbia, Canada

**DOI:** 10.1371/journal.pone.0210129

**Published:** 2019-01-10

**Authors:** Laura MacDougall, Kate Smolina, Michael Otterstatter, Bin Zhao, Mei Chong, David Godfrey, Ali Mussavi-Rizi, Jenny Sutherland, Margot Kuo, Perry Kendall

**Affiliations:** 1 British Columbia Centre for Disease Control, Vancouver, BC, Canada; 2 School of Population and Public Health, University of British Columbia, Vancouver, BC, Canada; 3 Data Management and Stewardship Branch, British Columbia Ministry of Health, Victoria, BC, Canada; 4 Performance Measurement and Reporting, Provincial Health Services Authority, Vancouver, BC, Canada; 5 Office of the Provincial Health Officer, British Columbia Ministry of Health, Victoria, BC, Canada; British Columbia Centre for Excellence in HIV/AIDS, CANADA

## Abstract

**Introduction:**

British Columbia (BC), Canada declared a public health emergency in April 2016 for opioid overdose. Comprehensive data was needed to identify risk factors, inform interventions, and evaluate response actions. We describe the development of an overdose cohort, including linkage strategy, case definitions, and data governance model, and present the resulting characteristics, including data linkage yields and case overlap among data sources.

**Methods:**

Overdose events from hospital admissions, physician visits, poison centre and ambulance calls, emergency department visits, and coroner’s data were grouped into episodes if records were present in multiple sources. A minimum of five years of universal health care records (all prescription dispensations, fee-for-service physician billings, emergency department visits and hospitalizations) were appended for each individual. A 20% random sample of BC residents and a 1:5 matched case-control set were generated. Consultation and prioritization ensured analysts worked to address questions to directly inform public health actions.

**Results:**

10,456 individuals suffered 14,292 overdoses from January 1, 2015 to Nov 30, 2016. Only 28% of overdose events were found in more than one dataset with the unique contribution of cases highest from ambulance records (32%). Compared with fatal overdoses, non-fatal events more often involved females, younger individuals (20 to 29 years) and those 60 or older. In 78% of illegal drug deaths, there was no associated ambulance response. In the year prior to first recorded overdose, 60% of individuals had at least one ED visit, 31% at least one hospital admission, 80% at least one physician visit, and 87% had filled at least one prescription in a community pharmacy.

**Conclusion:**

While resource-intensive to establish, a linked cohort is useful for characterizing the full extent of the epidemic, defining sub-populations at risk, and patterns of contact with the health system. Overdose studies in other jurisdictions should consider the inclusion of multiple data sources.

## Introduction

Overdose deaths involving opioids have substantially increased in North America and Europe since the turn of the millennium[1, 2]. The opioid-related overdose epidemic in British Columbia (BC), Canada (population 4.5 million) was declared a public health emergency in April 2016 following a rapid escalation in the number of illicit drug deaths in the province. From January to April 2016, an average of 73 people per month were dying from illicit drug overdoses, an increase of 70% over 2015 values, and 140% over 2014 values [3]. Among those aged 19 to 29 years, deaths due to illegal drug overdose surpassed cancer as the leading cause of mortality [3, 4]. The declaration of the emergency precipitated the formation of a provincial overdose response structure, which sought to coordinate the efforts of law enforcement and health. Several Task Groups were created to focus efforts on specific response activities: 1) escalation of the Take Home Naloxone program, 2) delivery of addictions treatment, 3) supervised consumption and drug checking, 4) public health surveillance, 5) communications, and 6) logistics.

Provincial public health surveillance infrastructure was rapidly set up to monitor the incidence and the demographic and geographic patterns of overdose in near real-time. This approach used data from the BC Ambulance Service, the BC Coroner’s Service, case-based and syndromic reporting from emergency departments, and calls to the Provincial Drug and Poison Information Centre. However, it was anticipated that these data streams, while essential to support timely situational awareness, would be insufficient to provide a detailed understanding of the population at risk, or to more precisely target intervention efforts. Linkage of individual data sources to one another would enable a better understanding of a person’s experience with the health system at the time of an overdose (e.g., the proportion of individuals suffering fatal overdose who called an ambulance), which could then be used to inform peer and public health education campaigns. Additional linkages to a person’s historic health data could be used to assess the use of health services prior to overdose (leading to targeted locations for intervention), understand health and socioeconomic profiles for those experiencing overdose, and reveal patterns of comorbidities and prescription medication use leading to overdose (which could then be used to identify risk and protective factors).

In collaboration with regional health authorities, the BC Ministry of Health, BC ambulance services, and the BC Coroner Service, the BC Centre for Disease Control (BCCDC) led the development of the Provincial Overdose Cohort (hereafter, the Overdose Cohort). The Overdose Cohort links public health surveillance and administrative healthcare data for individuals who have experienced opioid-related overdoses (non-fatal and fatal) from both illicit and prescription drug use. The immediate purpose of the Overdose Cohort was to meet the information needs described above; in the long term, the Overdose Cohort will also help evaluate the impact of policy and public health interventions used to respond to the overdose crisis.

In addition to identifying and integrating specific data, defining the data linkage strategy and developing case definitions, a key part of this work was to develop a novel data governance strategy. Given the public health emergency, there was a need to ensure that scarce public health analytic resources were targeting critical questions identified by stakeholders, that analytic efforts were coordinated with minimal duplication, that analysis was collaborative, and that standardized definitions and methods were applied.

Here, we describe the process of construction, oversight and use of the Overdose Cohort. Specifically, the integration of data sources, data linkage strategy, case definitions, and data governance model are discussed. The characteristics of the resulting cohort are also presented along with its data linkage yields and a summary of the case overlap among data sources.

## Methods

### Data sources

Nine data sources were linked at the patient level to form the Provincial Overdose Cohort. Individuals who experienced an overdose event were identified by data sources 1–7 below; five years of health history on these individuals from BC’s single payer health system were appended from data sources 5–8, and data source 9 was used to identify status First Nations people.

1. BC Ambulance Service Patient Care Reports (PCR). Contains information about the time and location of an overdose event, demographic information about the patient, and details of the paramedic’s assessment, treatment and transportation of patients, including whether or not naloxone was administered by paramedics.
2. Drug and Poison Information Centre (DPIC). Contains information about calls to DPIC from the public or from medical personnel for clinical advice on poisoning management. It includes date and time of call, age and sex of the patient, postal code and city of call origin, drugs associated with the call, route of drug administration, symptoms and outcome.
3. BC Coroner’s Service (BCCS). The coroner investigates all accidental and undetermined illicit drug overdose deaths in British Columbia. Data contains information about date of death/injury, age group, occupation and gender of decedent, location of death, location of residence of the decedent, cause(s) of death, frequency of drug use and route of administration, toxicology results, and history of recent incarceration.
4. Case-based reporting by Emergency Departments (ED). Emergency departments (EDs) in three of five BC Health Authorities (Interior Health, Island Health, and Northern Health) completed paper-based reporting to public health for each case of opioid-related drug overdose they treated. These forms contained data on patient demographics, overdose setting, circumstances of overdose (e.g. use alone), frequency of drug use, drugs taken and route of administration, naloxone received. All EDs in Interior Health, Vancouver Island Health Authority, and Northern Health participated. Overdose notifications from the two remaining health authorities in BC were included as part of NACRS reporting (below).
5. National Ambulatory Care Reporting System (NACRS). Captures all levels of ambulatory care within BC, including visits to the EDs. In 2016, NACRS Level 2 was implemented in 29 EDs, which accounted for 67% of all ED visits in the province, and close to 100% of EDs in the urban health authorities of Vancouver Coastal and Fraser [5]. Diagnostic coding uses an abbreviated version of International Classification of Disease (ICD) 10 codes, the Canadian Emergency Department Diagnoses Shortlist (CED-DxS); not all ICD10 codes are included.
6. Discharge Abstract Database (DAD). Captures discharges, transfers and deaths occurring in acute care hospitals in BC. Information about a patient stay includes patient demographics, timing of the stay, location of service, provider type, and information on health conditions and procedures relevant to the stay. Diagnostic coding uses ICD10 codes.
7. Medical Services Plan (MSP). Contains records of all fee-for-service provider visits billed to the province’s universal health insurance program. This excludes services by providers on alternative payment plans. Providers are classified as physicians or supplementary benefit practitioners (e.g. physiotherapists). Information provided about a visit includes patient demographics, service date, service location, provider type, and reason for the visit using ICD9 coding scheme.
8. PharmaNet (PNET). Contains records of all prescription dispensations in the province of BC. Information about a dispensation includes patient demographics, geographic location of dispensation, type of prescriber, drug specific information, formulation and dose.
9. First Nations Client File (FNCF). FNCF is a cohort of B.C.-resident First Nations people registered under the Indian Act, and their unregistered descendants born after 1986 for whom entitlement-to-register can be determined, based on their BC Ministry of Health Personal Health Number (PHN). The First Nations Client File is the product of a record linkage between an extract of the Indigenous & Northern Affairs Canada (INAC) Indian Registry and the BC Ministry of Health Client Registry, which represents all British Columbians registered for provincial health insurance under its single-payer system. The FNCF cannot capture ‘Aboriginal identity’ which includes Metis and Inuit peoples nor self-identified First Nation people not registered under the Indian Act.

### Case definitions

#### Overdose event

Given that individual data sources lacked a common coding scheme, multiple case definitions were required for the identification of overdose events. Definitions focused on specificity rather than sensitivity, in an effort to accurately identify both the population at risk and the risk factors for overdose and overdose death. In as much as possible, definitions were constructed to capture *opioid-related* overdose events and deaths; however, different aspects were used to define an opioid-related overdose across the datasets, including naloxone administration, clinical judgement and ICD administrative codes. Except for deaths, opioid-related overdoses included those due to prescription medications. Unlike other data sources, overdose deaths also included some deaths due to illicit drugs other than opioids.

A case was defined as any person experiencing at least one overdose in British Columbia between 1 January 2015 and 30 November 2016, based on the following source-specific case definitions:

Ambulance Attended Opioid Drug Overdoses: Ambulance-attended events recorded in the BCEHS Patient Care Record for which patients were coded as receiving naloxone by paramedics.

Opioid-associated Call to DPIC: Any call with an AAPCCGenCode indicative of an opioid, with or without the involvement of other drugs/alcohol: 37701–05, 37707–8, 37784, 41700, 72700, 72702, 72704, 77810, 200625, 200628, 200630, 200638, 201063, 201131.

Case-based reporting by Emergency Departments: Any visit to an ED in a participating health authority between June 5^th^, 2016 and March 4th, 2017 where a physician's assessment of clinical symptoms indicated an opioid overdose, regardless of the patient’s self-reported drug use.

Illicit Drug Overdose Deaths: Overdoses involving street drugs (heroin, cocaine, MDMA, methamphetamine, etc.), medications that were not prescribed to the deceased, combinations of the above, with prescribed medications, and those overdoses where the origin of drug is not known. Both open investigations (toxicology pending) and closed cases were included.

MSP: A case was defined by the following International Classification of Diseases, version 9 (ICD-9) codes in the primary diagnostic field: 965.0 –poisoning by opiates and related narcotics, E850.0 –accidental poisoning by opiates and related narcotics.

DAD: A case was defined by the following ICD-10 codes in the primary discharge diagnosis field: T40.0 –poisoning from opium, T40.1 –poisoning from heroin, T40.2, T40.3, T40.4, T40.6 –poisoning from other opioids

NACRS: ICD-10 codes representing opioid overdose that are used in the Canadian Emergency Department Diagnoses Shortlist (CED-DxS) were included. A case was defined where the ICD-10 codes T40.1 or T40.6 were present in the ED discharge diagnosis field.

#### Overdose episode

Records were aggregated to construct groupings of events deemed to be related, i.e. part of a single overdose. In each data source, records were grouped for an individual that were determined, based on available variables, to be a single healthcare encounter (e.g., one hospital stay, one visit to ED or physician). An overdose episode was defined as one or more encounters, each separated by less than 24 hours based on time of event, with at least one encounter meeting our case definitions for overdose. The interval of 24 hours was chosen based on the limitations of several source datasets, which contained event dates but not exact times. An overdose episode was considered fatal if it included a BCCS record for illicit drug death; otherwise, the episode was assumed to be non-fatal.

#### Reference cohort

In order to provide a pool to select control cases as well as to serve as a reference source of demographics, risk factors, and healthcare use patterns in the general BC population, a Reference Cohort was constructed. This comprised a 20% random sample, based on the size of the 2016 BC population, of individuals present in the Ministry of Health’s Provincial Client Roster between 1 January 2015 and 30 November 2016. Individuals were flagged in the Reference Cohort if they were also present in the Overdose Cohort.

For comparison of health outcomes, a Matched Cohort was also generated, where individuals from the Overdose Cohort were matched 1:5 without replacement with individuals in the Reference Cohort (excluding overdose cases) based on birth year, sex, and local health area of residence. The matching algorithm selects the closest match for each case among the available controls and then repeats until five controls are matched for every case. Birth year match was broadened if exact matches did not exist; in total 99.5% (N = 52023/52275) of controls met our match criteria exactly, with the remaining matched with birth year within ± 4 years.

#### Health data

**Fig 1:**
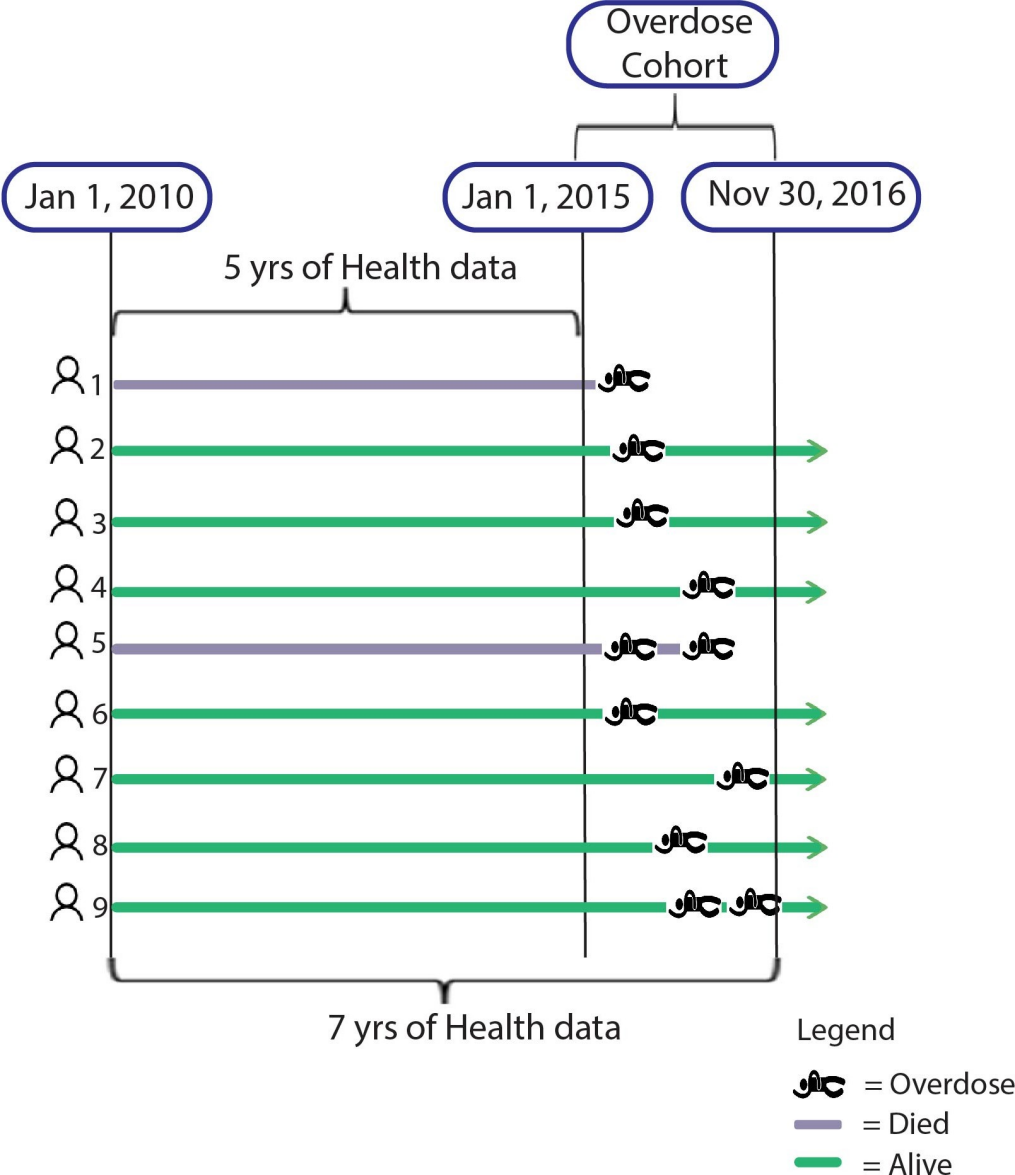
Schematic of health services data appended to individuals in the Provincial Overdose Cohort. Health records for each patient were collected from Jan 1, 2010 until the end of the study period, November 30, 2016. Each patient in the cohort had a minimum of 5 years of historic health data available; patients overdosing late in the cohort recruitment period had more than 5 years available. A small amount of health data post-overdose was available; this will grow with annual data refreshes in future years.

Five years of health service use data were appended to cases in the Overdose and Reference Cohorts according to the schema in Fig 1. Health Data from data sources 5–8 were requested from 1 Jan 2010 to 30 Nov 2016 or the most recently available at the time of data extraction. Each patient in the cohort thus had a minimum of 5 years of historic health data available; patients overdosing late in the cohort recruitment period had more.

#### Covariates

Social and Material Deprivation indices were developed using CensusPlus 2011 data, and are defined by levels of education, employment, income, living alone and lone parenting. These measures were assigned to cases and controls based on their geography of residence (local health area)[6]. Health Authority of the overdose event was assigned based on the incident location, or when missing by location of death or residence, in that order. Setting (e.g. rural, remote) was defined by the BC Community Health Service Area (v2017.1) urban/rural classification system. The number of health care visits (ED visit, hospital admission, physician visit, prescriptions) were based on the 12 months prior to first recorded overdose with a matched time period used for controls. The number of prescription encounters is based on prescriptions/per person/per day and represents a count of distinct community pharmacy encounters not individual prescriptions. Chi Square tests were used to assess significant differences among covariates between cases and controls and between fatal and non-fatal overdoses.

### Data acquisition, linkage, and structure

As part of the public health emergency declaration, the Provincial Medical Health Officer of BC issued Public Health Orders under the BC Public Health Act for the acquisition of data from BC Emergency Departments, the BC Ambulance Service and the BC Coroner’s Service that would otherwise have been unavailable. Under the BC Public Health Act, through an emergency declaration, additional powers are granted for Medical Health Officers and the Provincial Health Officer to collect necessary data even if that requires overriding other existing acts e.g. Coroner’s Act and provisions of the BC Freedom of Information and Protection of Privacy Act. An information sharing agreement was developed between the BC Centre for Disease Control, the BC Ministry of Health and First Nations Health Authority to link the Provincial Overdose Cohort with administrative health datasets (NACRS, DAD, MPS, PNET) and the First Nations Client File, as well as to generate the Reference Cohort.

Public Health Reporting Data Warehouse (PHRDW) patient matching service was used to link patients in datasets 1–4. The PHRDW service uses a series of deterministic and probabilistic linkage algorithms to match patients using first name, middle name, last name, date of birth, sex and provincial health number (PHN), if available. Where possible, PHNs could be recovered from the larger pool of patients in the service to enhance the potential to match them with administrative data. The validity of this method was assessed upon initial development [7] and as new data sources are introduced. The same service has been successfully utilized in other studies [8].

**Fig 2:**
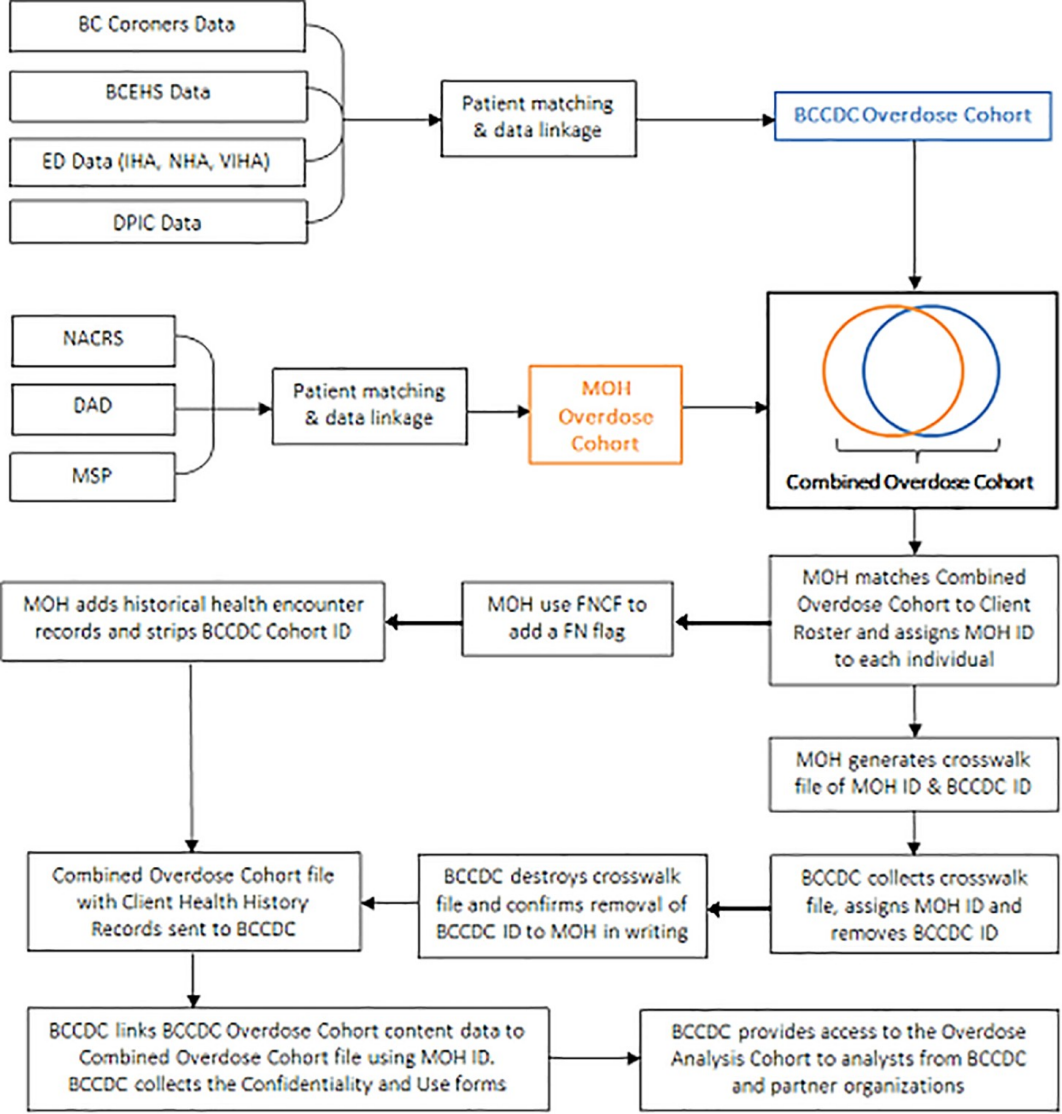
Data linkage diagram. BCEHS = BC Emergency Health Services; ED = Emergency Department; DPIC = Drug and Poison Information Centre; NACRS = National Ambulatory Care Reporting System; DAD = Discharge Abstract Database; MSP = Medical Services Plan; MOH = Ministry of Health.

Additional overdose cases meeting the ICD-9 and -10 definitions for NACRS, DAD and MSP were identified by the Ministry of Health, and included in the Overdose Cohort if it could be established, via a deterministic link on PHN, that they were new cases. Deterministic linkage based on PHN was subsequently performed to append the five-year health data from MSP, DAD, NACRS and PNET to all individuals in the Overdose Cohort and Reference Cohort (Fig 2). Linkages were performed through the use of study identifiers and a crosswalk file such that neither party could re-identify individuals following construction of the final file. A de-identification process removed names, dates of birth and PHNs. Patient home addresses were aggregated to Local Health Area and, where available, overdose locations were aggregated to the census dissemination area level. Where appropriate, times were rounded to the nearest hour in the final file to prevent re-identification of specific events.

Data was stored in a relational database and accessed through SAS Enterprise Guide. Subsets of data used for analysis were stored on the SAS server as SAS datasets. Several master files were created based on cleaned and re-structured source data files. The first comprised all patients and their demographic/geographic characteristics, the second contained all healthcare encounters for each individual and the third contained all overdose episodes for each individual.

### Data governance

In order to ensure that the analytic questions being asked of the cohort were aligned with the information needs of those delivering public health interventions, members of the Surveillance Task Group held consultations within each of their organizations. Submissions were also solicited from each of the five other Task Groups in the response structure. The resulting analytic themes were prioritized by each organization according to defined criteria and a composite score applied. Data stewards from each organization reviewed the list of analysis themes to confirm that the question/methods applied did not violate provisions of any data sharing agreements in place. Individuals with content knowledge or analytic expertise from all member organizations were pooled and assigned to an analytic theme in groups of five to seven according to skillset and interest. More analysis themes than resources were identified; the highest priority themes were investigated first.

Analysts worked in a common secure SAS server environment with the ability for remote access through Citrix. Each analyst signed a confidentiality acknowledgement, and data was not permitted outside the analytic environment. Analysis teams worked to refine analytic questions, develop methodology, generate and review output. All teams were required to document any new derived variables created and share analytic code with other teams to promote standardization. Results were summarized, presented to stakeholders and shared in an on-line results repository available to members of the provincial response structure.

A consultation with the University of British Columbia ethics board confirmed that the analyses being conducted were considered part of public health investigation and response under the Provincial Public Health Act, rather than research, and that no ethics approvals were required.

**Fig 3:**
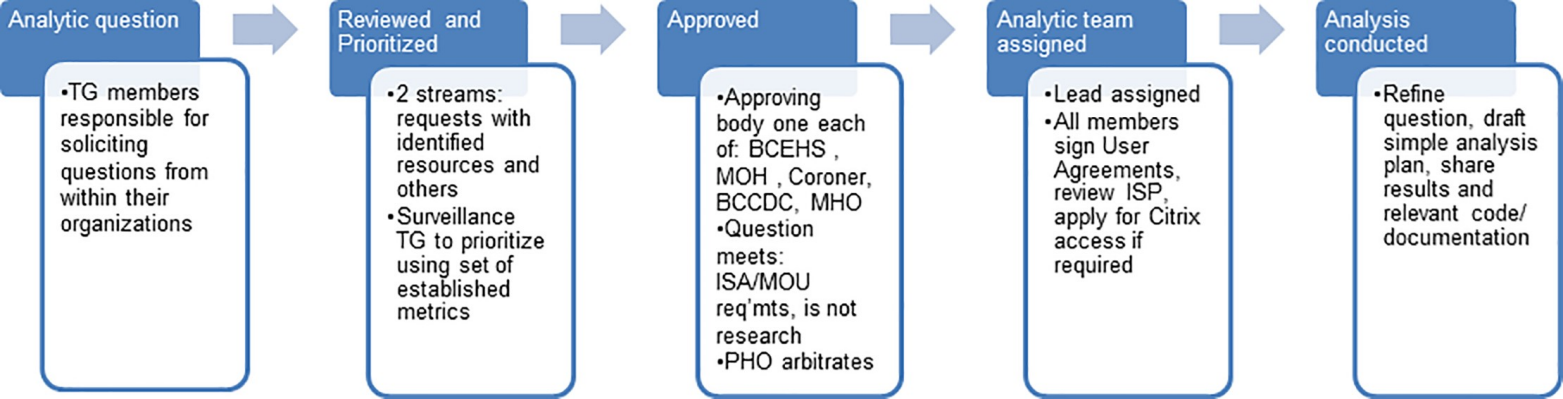
Governance of the Overdose Cohort. BCEHS = BC Emergency Health Services; MOH = Ministry of Health; BCCDC = BC Centre for Disease Control; MOH = Medical Officer of Health; PHO = Provincial Health Officer.

Steps in the governance process are summarized in Fig 3.

## Results

### Priority analysis themes and timeline

Following consultation and prioritization, a list of 10 analysis themes were generated in priority order:

1. Factors related to death versus survival
2. Description of overdose in First Nation populations
3. Prescribing patterns prior to overdose
4. Risk factors for overdose and repeat overdose
5. Factors associated with overdose among those engaged in Opioid Agonist Therapy (OAT)
6. Patterns of health services utilization
7. Description of overdose by housing type
8. Description of areas of lower OAT penetration across the province
9. Effect of recent incarceration
10. Evaluation of long-term outcomes of overdose patients

The execution of data sharing agreements, definition of variables to be included and extraction, linkage and transfer efforts took eight months to complete; analysts spent a further two months working to clean, and restructure the data for analyses.

### Linkage yield and overlap of data sources

**Fig 4:**
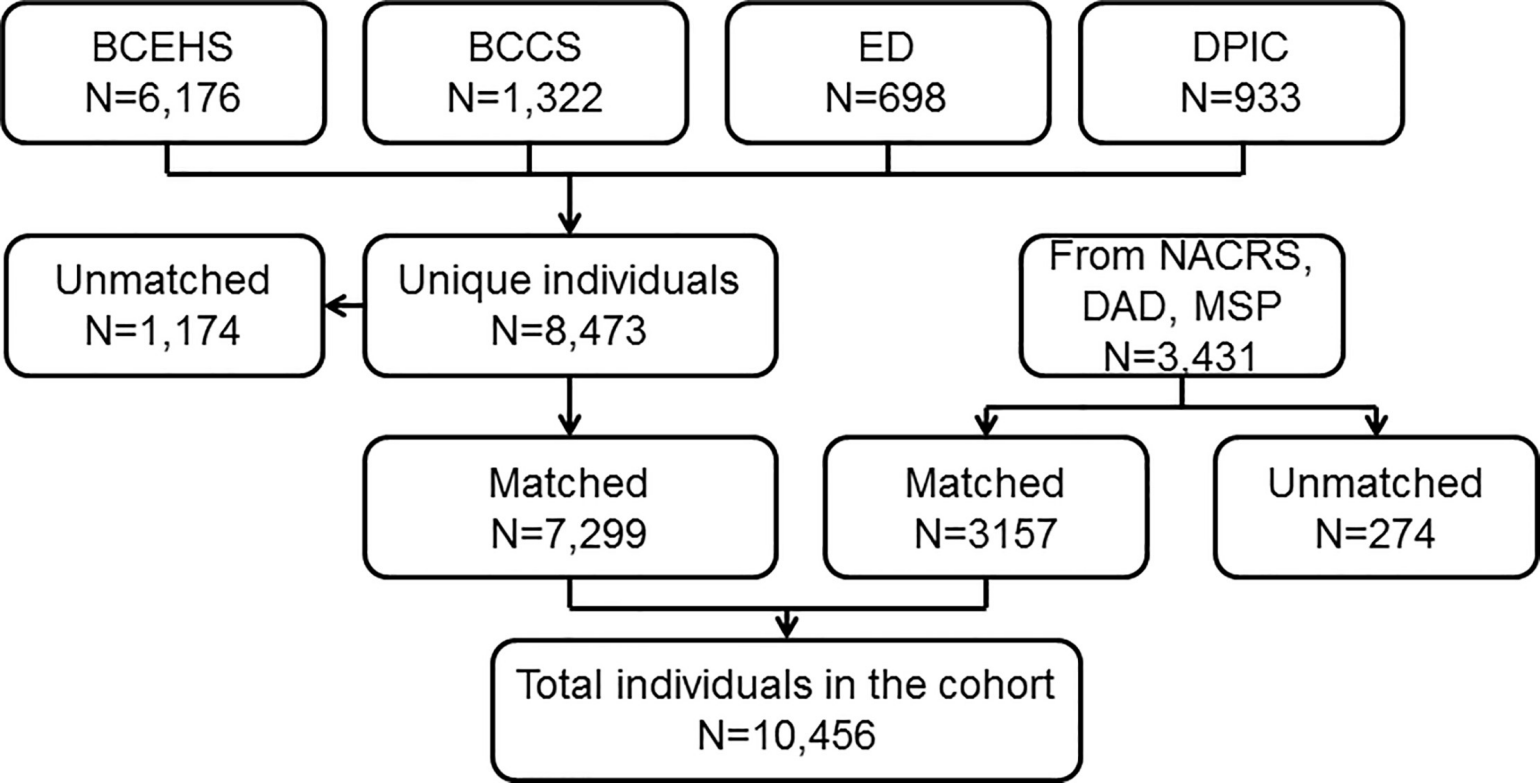
Linkage diagram, Overdose Cohort, January 1, 2015-Nov 30, 2016. BCEHS = BC Emergency Health Services; BCCS = BC Coroner’s Service; ED = Emergency Department; DPIC = Drug and Poison Information Centre; NACRS = National Ambulatory Care Reporting System; DAD = Discharge Abstract Database; MSP = Medical Services Plan.

Fig 4 depicts the linkage of individuals within the cohort. BCCDC-held databases (BCEHS, BCCS, DPIC, ED) were linked and de-duplicated producing 8,473 unique individuals. Of these, 14% of individuals (1,174/8,473) were unable to be matched to health data due to insufficient demographic information. DPIC records were the most likely to have insufficient demographics (70%), followed by ambulance records (8.3%), emergency department reports (2%) and Coroner reports (2%). A further 3,431 individuals were identified through opioid-specific ICD records in health administrative databases (DAD, NACRS, MSP). 10,456 individuals were ultimately included in analyses.

**Fig 5:**
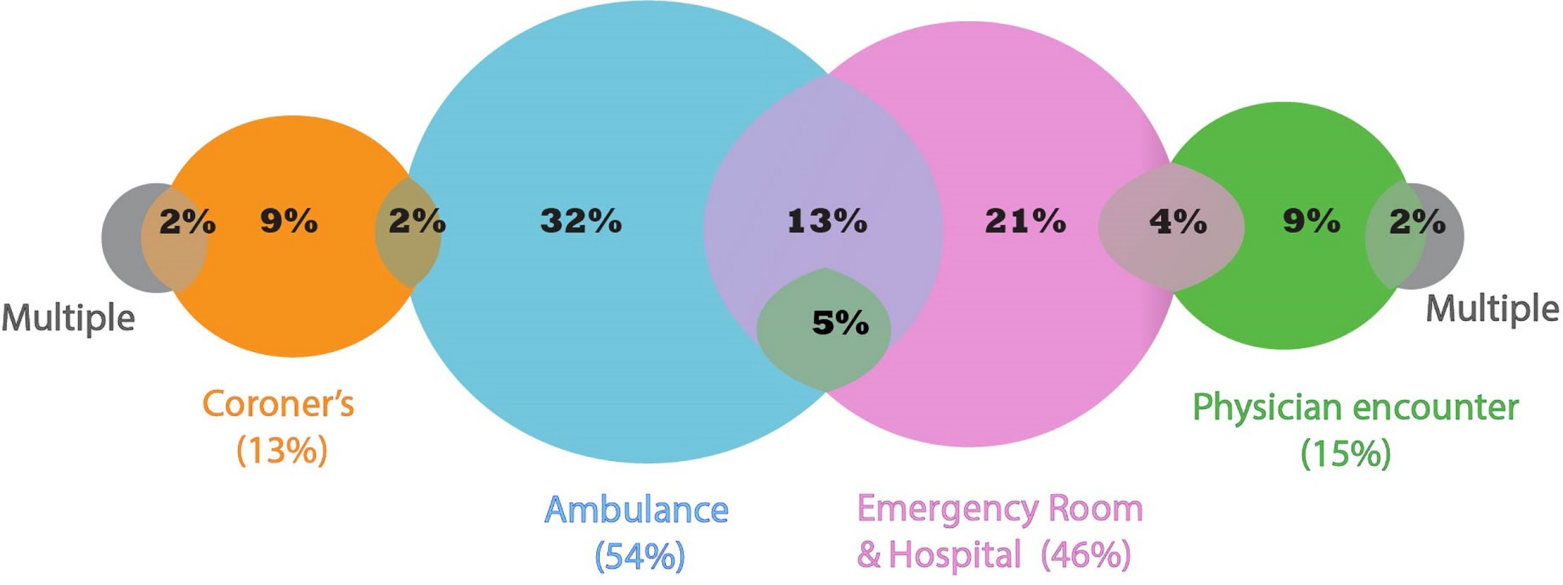
Case overlap between overdose data sources 1–7. The figure accounts for data sources used to identify 99% of all overdose events. The remaining 1% represents those events that appeared in multiple data sources. Bubbles indicate distinct data sources used to identify overdose events. Colours are used to differentiate between different data sources as well as highlight cases that were identified in more than one data source. Percentages in brackets indicate the total portion of all events identified through that specific data source. Percentages inside bubbles indicate the portion of all events identified through one or multiple data sets. The green bubble in the centre represents 5% of cases that were identified in three data sources: ambulance records, acute care, and physician encounters. The ‘Emergency Room and Hospital’ bubble includes records identified by either ED, NACRS, DAD or DPIC.

A given overdose event may have been present in multiple source datasets. For example, a patient that was transported by ambulance to the emergency department where they later died might be represented in BCEHS, ED, NACRS and Coroner’s datasets. Fig 5 displays the amount of overlap between data sources in this cohort. Only 28% of overdose events were found in more than one dataset over the study period. The unique contribution of cases was highest from ambulance records (32%), followed by acute care records (ED, NACRS, DAD) (21%); MSP and BCCS each identified 9% of unique records.

### Cohort characteristics

14,292 overdose events occurred over a 23 month period between January 1, 2015 and November 30, 2016 (Table 1). The vast majority (91%) of events (12,970/14,292) were non-fatal. There were notably more males in the Overdose Cohort compared with the Reference Cohort, with a higher proportion of men noted among fatal versus non-fatal overdoses (80% and 66%, respectively). The Overdose Cohort also included a higher proportion of individuals aged 20 and 49 years old than those in the general population. Overdoses happened across BC, in both urban and rural settings, with case distribution roughly equivalent to the population distribution across these regions (Table 1). Rates of fatal overdose were high and did not vary much geographically (12.9–16.0/100,000 population). 24% of fatal overdoses and 21% of non-fatal overdoses were in the most socially deprived category, compared with only 14% of the Reference Cohort.

**Table 1:** Demographic characteristics of the overdose and reference cohort.

	Overdose Cohort						Reference Cohort
	Non-fatal Overdoses			Fatal Overdoses			
	Individuals N (%)	Events N (%)	Event rate per 100,000	Individuals N (%)	Events N (%)	Event rate per 100,000	Individuals N (%)
**N**	9134 (100)	12970 (100)	143.2	1322 (100)	1322 (100)	14.6	998739 (100)
**Sex**							
Male	5869 (64)	8599 (66)	191.3	1059 (80)	1059 (80)	23.6	494731 (50)
Female	3265 (36)	4371 (34)	95.8	263 (20)	263 (20)	5.8	503939 (50)
Unknown	0 (0)	0 (0)	-	0 (0)	0 (0)	-	69 (0)
**Age group**							
0 to 14 years	54 (1)	54 (0)	4.1	0 (0)	0 (0)	-	141607 (14)
15 to 19 years	380 (4)	462 (4)	87.7	35 (3)	35 (3)	6.6	53554 (5)
20 to 29 years	2276 (25)	3403 (26)	276.7	271 (20)	271 (20)	22.0	131334 (13)
30 to 39 years	2223 (24)	3331 (26)	273.0	353 (27)	353 (27)	28.9	136232 (14)
40 to 49 years	1663 (18)	2410 (19)	198.1	311 (24)	311 (24)	25.6	131991 (13)
50 to 59 years	1388 (15)	1909 (15)	141.5	290 (22)	290 (22)	21.5	148082 (15)
60+ years	1149 (13)	1400 (11)	63.7	62 (5)	62 (5)	2.8	255932 (26)
Unknown	1 (0)	1 (0)	-	0 (0)	0 (0)	-	7 (0)
**Health Authority**							
Interior	1331 (15)	1763 (14)	123.8	198 (15)	198 (15)	13.9	160093 (16)
Fraser	3478 (38)	4915 (38)	145.2	472 (36)	472 (36)	13.9	361236 (36)
Vancouver Coastal	2365 (26)	3764 (29)	168.7	358 (27)	358 (27)	16.0	246041 (25)
Vancouver Island	1239 (14)	1583 (12)	106.5	197 (15)	197 (15)	13.2	164098 (16)
Northern	600 (7)	813 (6)	152.6	69 (5)	69 (5)	12.9	59905 (6)
Unknown	121 (1)	132 (1)	-	28 (2)	28 (2)	-	7366 (1)
**Urbanicity**							
Metro	5028 (55)	5516 (43)	182.9	737 (56)	619 (47)	20.5	555808 (56)
Urban/Rural	3088 (34)	2806 (22)	59.7	439 (33)	446 (34)	9.5	316064 (32)
Rural	800 (9)	557 (4)	53.1	106 (8)	91 (7)	8.7	105716 (11)
Remote	97 (1)	53 (0)	18.1	12 (1)	14 (1)	4.8	13785 (1)
Unknown	121 (1)	4038 (31)	-	28 (2)	152 (11)	-	7366 (1)
**Social Deprivation**							
Q1 (most deprived)	2208 (24)	3009 (23)	237.1	338 (26)	282 (21)	22.2	140650 (14)
Q2	919 (10)	787 (6)	85.6	130 (10)	121 (9)	13.2	103916 (10)
Q3	1122 (12)	938 (7)	79.8	165 (12)	169 (13)	14.4	132699 (13)
Q4	1439 (16)	1169 (9)	69.7	177 (13)	166 (13)	9.9	184379 (18)
Q5 (least deprived)	3325 (36)	3029 (23)	75.4	484 (37)	432 (33)	10.8	429729 (43)
Unknown	121 (1)	4038 (31)	-	28 (2)	152 (11)	-	7366 (1)
**Material Deprivation**							
Q1 (most deprived)	226 (2)	164 (1)	71.9	28 (2)	29 (2)	12.7	25253 (3)
Q2	630 (7)	445 (3)	49.6	81 (6)	65 (5)	7.2	98612 (10)
Q3	3099 (34)	2780 (21)	105.1	429 (32)	396 (30)	15.0	288634 (29)
Q4	2413 (26)	3024 (23)	162.1	338 (26)	310 (23)	16.6	206711 (21)
Q5 (least deprived)	2645 (29)	2519 (19)	73.6	418 (32)	370 (28)	10.8	372163 (37)
Unknown	121 (1)	4038 (31)	-	28 (2)	152 (11)	-	7366 (1)
**Number of overdoses per person**							
1	7198 (79)			1135 (86)			
2	1196 (13)			122 (9)			
3+	740 (8)			65 (5)			

Compared with fatal overdoses, non-fatal events tended to occur more commonly in females (36% vs. 20%; χ^2^ = 129.80, p < 0.001) and among younger age groups (median [25th percentile-75th percentile] age, fatal 40.1 [30.6–50.6] years; non-fatal: 38.1 [28.3–51.5] years; χ^2^ = 138.56, p < 0.001). (Table 1). Fatal and non-fatal overdoses also differed in terms of the individual’s health authority of residence (χ^2^ = 12.37, p = 0.033) and number of previous overdoses (χ^2^ = 36.11, p < 0.001), but only slightly by level of material deprivation (χ^2^ = 10.57, p = 0.058) and social deprivation (χ^2^ = 10.31, p = 0.068) quintiles, and not at all by the urbanicity of their place of residence (χ^2^ = 6.32, p = 0.181) (Table 1).

In 78% of illegal drug deaths, there was no ambulance response associated with the fatal overdose episode. In addition, most fatal events were not treated in the emergency department or hospital prior to death—only 12% were seen in emergency departments and 8% were admitted to hospital for any reason. A greater proportion of fatal than non-fatal overdoses happened in private residences (62% vs 49%).

Previously reported information on the circumstances of drug use was available from a subset of cohort cases identified through case-based reporting of opioid overdoses in the Emergency Departments of three BC Health Authorities [9]. A total of 1,510 visits by 1,262 patients were notified from 47 hospitals and community health centres between June 5, 2016 and March 4, 2017; characteristics were not significantly different when analyses were restricted to cases notified between Jan 1, 2015 and Nov 30, 2016. Information about whether the patient reported being alone at the time of the overdose was missing for 32% of visits; of those with information, 37% indicated using alone[9]. Among the 1,087 individuals where address information was reported, 17% reported no fixed address [9]. Polysubstance use was common; 45% of all visits where a substance was noted (n = 1362) involved self-reported use of two or more different types of drugs [9]. Self-reported heroin, used alone or in combination with other drugs, accounted for 50% of visits [9]. Whether reported alone or in combination with other drugs, use of one’s own prescription opioid medication was rare (7% of overdose visits) as was use of an opioid prescribed to someone else (13% of overdose visits)[9].

Some individuals in the overall cohort had overdosed multiple times during the course of the study period (Table 1). However, the vast majority, especially those who died, had only a single overdose event recorded throughout the 23 months of the cohort (Table 1). When restricting individuals to those with at least one year in which to experience subsequent overdose, 71% (578/811) of those who died still suffered only a single event.

Clients’ health system utilization prior to first overdose event indicated a high connectivity to services (Table 2). In the 12 months prior to their first recorded overdose, 60% of individuals had at least one ED visit, 31% at least one hospital admission, 80% at least one physician visit, and 87% had filled at least one prescription in a community pharmacy; in all cases, health service utilization was greater among people who overdosed than among matched controls. Table 2 shows that, compared to those who survived, proportionately fewer individuals who died of overdose had any visit to ED, hospital or community physician, or had any prescription, in the year prior to overdose (P < 0.001 for each comparison). The distributions of individuals by number of visits or prescriptions was similar between those with fatal vs non-fatal overdose, except that the former tended to have more individuals with no visits or prescriptions and fewer individuals with many visits or prescriptions (P < 0.05 in for each comparison).

**Table 2:** Health services utilization prior to overdose.

	Overdose Cohort				Matched Controls	
	Individuals Surviving Overdose		Individuals not Surviving Overdose			
	N	%	N	%	N	%
**Total individuals**	9134	100	1322	100	52275	100
Services accessed in year prior to first overdose*						
Any ED visit	5577	61	733	55	8990	17
Any hospital admission	2917	32	374	28	4910	9
Any physician visit (community practitioner)	7382	81	1005	76	37379	72
Any prescription (community pharmacy)	8010	88	1120	85	39669	76
Any ED, hospital or physician visit	8158	89	1125	85	38471	74
ED visits (any reason) in year prior to first overdose*						
0	3557	39	589	45	43285	83
1	1633	18	244	18	5886	11
2–5	2620	29	362	27	2813	5
6–10	781	9	86	7	242	0
11+	543	6	41	3	49	0
Hospital admission (any reason) in year prior to first overdose*						
0	6217	68	948	72	47365	91
1	1552	17	221	17	3886	7
2–5	1212	13	136	10	994	2
6–10	124	1	16	1	24	0
11+	29	0	1	0	6	0
**Physician visits (community practitioner, any reason) in year prior to first overdose**						
0	1752	19	317	24	14896	28
1	726	8	136	10	6751	13
2–5	1776	19	276	21	16648	32
6–10	1417	16	200	15	8272	16
11+	3463	38	393	30	5708	11
Prescriptions (any, community pharmacy) in year prior to first overdose*^						
0	1124	12	202	15	12606	24
1	605	7	119	9	6714	13
2–5	1341	15	180	14	12428	24
6–10	915	10	131	10	7262	14
11+	5149	56	690	52	13265	25

*12 months prior to first recorded overdose within the study period was used for cases with a matched time period for controls

^ the number of prescription encounters is based on prescriptions/per person/per day and represents a count of distinct community pharmacy encounters not individual prescriptions

## Discussion

The integration of seven distinct sources of data on overdose events provided a more complete understanding of the extent of the opioid crisis in British Columbia than any single dataset alone. Overdose deaths, which are an important indicator used in near real-time monitoring in most Canadian jurisdictions, represented only 13% of individuals overdosing over almost two years. Overdose deaths tended to occur more often in males and slightly older age groups than non-fatal overdoses and occurred among people who were less likely to have experienced a prior overdose within the time frame of the cohort, suggesting that a person’s first documented overdose is often fatal. Jurisdictions developing surveillance systems should consider the inclusion of ambulance and emergency room data in order to characterize and monitor a more complete spectrum of the population at risk, and to evaluate the impact of interventions on both fatal and non-fatal overdoses. The larger number of incidents generated by inclusion of additional data can also better support geo-temporal cluster detection and the development of more robust early drug warning systems.

While findings from the Overdose Cohort may offer insights into the development of surveillance systems, it should not be considered–in and of itself–either a form of surveillance or replacement thereof. The effort and complexity required to integrate multiple data sets comes at the expense of the timeliness required for public health action. Instead, the value of the Overdose Cohort lies in its ability to gain a deeper understanding of relevant risk and protective factors, characterize sub-populations at increased risk, and investigate the degree to which people are connected to health services, including treatment. As public health actions unfold in BC, and the cohort is refreshed annually, it also offers the ability to evaluate the health outcomes for patients who have suffered an overdose, to characterize long-term sequelae and to measure program impact.

Unlike other jurisdictions, where overdoses have tended to cluster in urban areas, the opioid overdose crisis is affecting all geographic areas of British Columbia, including urban, suburban, rural and remote communities. The BC Coroner has highlighted the high number of fatal overdoses happening in private residences [3], and non-fatal overdoses show similar patterns.

Based on a subset of overdoses that were identified through EDs, almost 40% of non-fatal opioid overdoses happened in people who self-reported using alone. A high degree of non-response for this item likely underestimates the proportion using alone, due to social desirability bias compounded by the fact that unconscious patients (those unable to respond to questions) may be more likely to have used alone. Fatal overdose events mainly occurred without connection to any health services, including an ambulance call, supporting the contention that those who die are also using illegal drugs alone. Harm reduction messages in BC have therefore focused on strategies that encourage use with others, with at least one person sober enough to call 911, and if drug use does occur alone that doors are left unlocked so that help can be provided quickly if required.

Despite the fact that 70% of fatal overdose episodes had no associated ambulance, ED or hospital encounter at the time of overdose, most people who overdosed–including those who died of overdose–have largely had contact with health services (ED, hospital, physicians) *prior* to their overdose event. This suggests potential opportunities for intervention and is consistent with previous studies in the United States and Australia that have found similarly high levels of ED and hospital use among people who use drugs [10–13]. Given the volume of overdoses occurring daily in BC, a strategy for identifying and triaging patients at highest risk into appropriate services should be considered. ‘Warm hand-offs’ have been studied [14] and piloted in some areas of the province (e.g., ED referrals to mental health and addictions services for people overdosing alone in private residences). Refinements to triage protocols can be informed by inferential and cluster-based modelling results from the cohort that identify risk factors for overdose and overdose death and sub-populations at particular risk.

Contextual data about the circumstances of drug overdose are not readily available through administrative data sources. Case-based surveillance of opioid overdoses in ED in some BC health authorities not only supplemented NACRS data in areas where hospitals did not report, but provided valuable baseline indicators of factors such as using alone, type of drugs taken, and frequency and location of drug use. However, collection of this information was ultimately unsustainable and was discontinued in the summer of 2017. Ongoing changes in contextual factors will be assessed through survey and/or qualitative means on a periodic basis; only basic information on overdoses will continue to be collected to ensure good geographic representation of overdoses in the cohort.

Case definitions sought to include opioid-related overdoses rather than all illegal drug overdoses in the cohort for two reasons. First, information from ED surveillance demonstrated that non-opioid drugs alone were reported in just 14% of overdose events clinically compatible with opioid overdose [9]. This suggests that while contamination of non-opioid drugs such as stimulants is happening, this is occurring at relatively low levels in BC. Second, a low percentage of non-opioid drugs (i.e., cocaine, methamphetamines) were found to be mixed with fentanyl during seizure and testing of street drugs in BC by law enforcement [15]. Overdose deaths provided by the BC Coroners Service did include some deaths due to illicit drugs other than opioids; this amounted to 1.8% of all overdose events in the cohort. We considered excluding deaths based on toxicology results, however events occurring later in the cohort would not have had the same opportunity to be excluded (since toxicology results were still pending), introducing a temporal bias. Additionally, a small amount of misclassification may exist due to the inclusion of suspected illicit drug deaths; suspected cases are based on preliminary circumstances and may ultimately change to a different cause of death once the coroner investigation is concluded. Examinations of prescription-opioid related deaths in BC has shown that these constitute a relatively small proportion of all overdose deaths and have been stable over the past decade [16, 17].

Due to the nature of integrating multiple administrative datasets collected for a different primary purpose, the case definitions used could not be completely harmonized and naturally reflect the diversity in coding processes and practices among the organizations contributing data. One specific limitation pertains to the capture of naloxone administrations by BCEHS. Since 2012, BC provincial programs have been distributing no-cost Take Home Naloxone kits to individuals at risk of overdose or likely to witness and respond to an overdose. In September 2016, naloxone was delisted in BC, meaning that prescriptions were no longer required. As Take Home Naloxone programs subsequently increase their distribution, more naloxone will be administered to overdose victims by by-standers [18]. Bystander naloxone administrations are not being captured in ambulance records and thus these events are not identifiable as opioid-related overdoses. The extent to which these overdoses are picked up by other sources (e.g. ED, NACRS, DAD) is unknown. As BCEHS deploys a new information system in the next few years, they will begin documenting naloxone administered by any provider, including fire, ambulance, enforcement and by-standersin order to reduce opportunities for misclassification.

## Conclusion

The development, oversight and analysis of the Provincial Overdose Cohort marked a fundamental shift in the way that BC manages public health data assets. It represents a departure from the model where one agency holds, manages and analyses data with input from stakeholders, to an environment where teams of analysts from all stakeholder agencies access a common data platform and jointly conduct work that has been collectively prioritized by the broader public health community. New analyses will continue to be done based on periodic re-prioritization of knowledge gaps, particularly as additional data sources (e.g., provincial incarcerations) are added. Assembly of an overdose cohort, while resource-intensive to establish, has proven a useful tool for characterizing the full extent of the opioid crisis, defining sub-populations at risk, identifying risk factors, and patterns of contact with the health care system. Importantly, the addition of non-fatal overdoses, particularly from ambulance sources, contributed to a more comprehensive understanding of the magnitude and distribution of overdose events. Similar approaches in data linkage and analysis could be deployed in other jurisdictions to support ongoing efforts to address the widespread overdose crisis.
